# Critical analysis of the liver cancer policies and programs in China: implications for international liver cancer control

**DOI:** 10.1186/s41256-025-00450-w

**Published:** 2025-10-02

**Authors:** Xianwen Chen, Junnan Shi, Yan Xue, Yunfeng Lai, Menghuan Song, Carolina Oi Lam Ung, Hao Hu

**Affiliations:** 1 State Key Laboratory of Mechanism and Quality Research in Chinese Medicine, Institute of Chinese Medical Sciences, University of Macau, Room 1050, E12 Research Building, Taipa, Macao SAR, China; 2 School of Public Health and Management, Guangzhou University of Chinese Medicine, Guangzhou, China; 3 Centre for Pharmaceutical Regulatory Sciences, University of Macau, Macao, Macao SAR, China; 4 Department of Public Health and Medicinal Administration, Faculty of Health Sciences, University of Macau, Macao SAR, China

**Keywords:** Cancer control, Liver cancer, Health policy, Health program, China

## Abstract

**Background:**

Liver cancer is among the top five causes of cancer death in 90 countries, with China accounting for a substantial proportion of the global burden. This study aimed to analyse the national liver cancer policies and programs in China.

**Methods:**

This study applied a documentary research method using the systematic READ approach. Six national official websites and one public policy database were searched. The document analysis was based on the WHO Health System's Six Building Blocks and the WHO's four modules of cancer control (prevention, early detection, diagnosis and treatment, and palliative care).

**Results:**

A total of 74 liver cancer-related policies and 8 programs published from 1 January 1984 to 31 March 2025 were included in this study. The analysis revealed that liver cancer management in China mainly focused on *Medical products and Technologies* (n = 46, 62.16%), and *Service Delivery* (n = 34, 45.95%) within the WHO health system building blocks. When it came to WHO cancer control four modules, most policies (n = 39, 52.70%) targeted Diagnosis and Treatment of liver cancer, followed by Prevention of liver cancer (n = 33, 44.59%). Additionally, 8 national programs were implemented to improve the prevention, diagnosis, and treatment of liver cancer. Before 2019, the emphasis of liver cancer prevention was primarily on hepatitis prevention and control. However, since 2020, the national-level programs aimed at preventing and controlling liver cancer emphasizing patient education and treatment for high-risk groups.

**Conclusions:**

China’s liver cancer control mainly focused heavily on prevention, diagnosis and treatment modules with special focus on medical products and technology, as well as service delivery. Currently, less attention has been given to the detection and palliative care of survivors. The control of liver cancer in China still requires further strengthening of the health system for implementation. Considering the continual increase in the burden of liver cancer, it is imperative for future efforts to develop a comprehensive national liver cancer strategy.

**Supplementary Information:**

The online version contains supplementary material available at 10.1186/s41256-025-00450-w.

## Background

Liver cancer, ranked among the top five causes of cancer death in 90 countries, is a significant public health problem worldwide [1–4]. Despite a sustained decline in age-standardized incidence and mortality rates for liver cancer in China since 2005, the country continues to bear the highest absolute global burden, accounting for approximately 50% of worldwide incident cases and cancer-related deaths [5, 6]. Current data indicate that China ranks fourth globally in age-standardized incidence rate and second in mortality rate [7], with hepatitis-related cases being the largest patient group [8–10]. According to the 2021 Global Burden of Disease (GBD) database, the age-standardize prevalence of liver cancer in China was 18.66 per 100,000 population, with an incidence rate of 13.82 per 100,000 population [11]. Furthermore, China’s age-standardized mortality rate for liver cancer was 12.09 per 100,000 population, nearly twice the global age standardized rate (6.13 per 100,000 population) [11]. Notably, China accounted for 37.94% of the global burden of disability-adjusted life years (DALYs) attributable to liver cancer, with a total burden of 4,890,023.03 (equivalent to an age-standardized rate of 343.7 per 100,000 population) [11]. Liver cancer also presents a significant burden in China [12, 13]. There is potential for liver cancer to become the next target for elimination through coordinated global efforts [14].

According to the World Health Organization (WHO), 30–50% of all cancer cases are preventable [15]. Another one-third of the cases could be managed through early detection and prompt treatment where resources permit, and the remaining advanced cases could be managed with palliative care [16, 17]. In alignment with this, the WHO launched a framework for establishing a national cancer control program (NCCP) in 2002, outlining four modules of cancer control: prevention, early detection, diagnosis and treatment, and palliative care [16]. This guideline also highlighted the key role of effective policies on a national level in addressing the cancer problem worldwide [16]. A systemic and comprehensive approach is required to develop an extensive system integrated with other programs and health system rather than a single program operating in isolation [15, 18, 19]. There is increasing global recognition that national policies or programs are crucial to addressing the cancer burden effectively and prioritizing and coordinating policies [20]. As of August 20, 2024, 131 countries (66.50%) have established national cancer plans or programs, according to the International Cancer Control Partnership (ICCP) [21].

The importance of policy-guided liver cancer control has been highlighted in international comparative studies [22–24]. In the national cancer control plans currently being implemented, the targeted measures for liver cancer prevention and control in various countries are mainly the prevention and control of viral hepatitis, especially hepatitis B prevention and hepatitis B screening [18]. The National Cancer Institute of the United States has also developed a comprehensive liver cancer control roadmap, and the Centers for Disease Control and Prevention conducted various programs or actions for liver cancer control [25]. China has issued a series of policies to decrease the cancer burden and has developed focused strategies on primary prevention (Hepatitis B vaccine, cancer surveillance, and behavior interventions) and secondary prevention (cancer screening programs) [26]. However, there is no integrated framework or roadmap for liver cancer specifically, and the national liver cancer control policies and programs have not been analysed systemically.

Hence, this study aimed to analyse the national liver cancer policies and programs in China. The findings were expected to serve as valuable points of reference for China and international policymaking on advancing the development of national strategies for liver cancer control and strengthening health system for liver cancer management.

## Methods

### Study design

This study applied a documentary research method by following a systematic procedure of READ approach [27]. There are four main steps to gain key information from the context of policy documents: (1) Ready the materials, (2) Extract data, (3) Analyse data, and (4) Distil findings [27].

### Data source

The official websites of relevant government departments and professional organizations (including (1) the State Council of the People’s Republic of China; (2) the National Health Commission of the People’s Republic of China; (3) the National Medical Products Administration; (4) the Chinese Center for Disease Control and Prevention; (5) the National Administration of Traditional Chinese Medicine; and (6) the National Healthcare Security Administration were searched for relevant information, reference publications, and databases describing national initiatives for liver cancer control. In addition, the search process was broadened to include target policies through a public document database *Beidafabao* (www.pkulaw.com/law). Policies were included if they were active or had been formally enacted by March 31, 2025.

### Search strategy

The search terms used in this study were a combination of the following terms in Chinese and English: gan (liver) OR ganai (liver cancer) OR ai (cancer) OR zhongliu (carcinoma); liver OR liver cancer OR cancer OR carcinoma. The inclusion and exclusion criteria of this study are presented in Table 1.

**Table 1:** Inclusion and exclusion criteria of document

Inclusion criteria	
1. Document types [27]	Official documents Policies or policy directives Strategies for sectors or specific health problems Official statements and declarations Official position papers Statistical surveys or publications Implementation documents Training manuals or work tools (booklets, clinical files, etc.) Midterm/final reports or evaluations Financial analyses, Operational plans, Project proposals, Funding requests Legal documents Laws Regulations Memorandums of understanding Cooperation agreements
2. Documents’ content	About the national liver cancer control initiatives, including: Prevention [28] Early detection [29] Diagnosis and treatment [30] Palliative care [31]
3. Time period	From inception to March 31, 2025
Exclusion criteria	
1. Language restrictions	Not English or Chinese
2. Document type	Other types of documents: working documents, region-specific documents, specific clinical or pharmaceutical guidelines, policy responses or interpretations
3. Scope	Focus on a specific region, not national document
3. Validity of the document	Invalidated documents

### Data screening and extraction

Two authors conducted the document search independently. Duplicates were then removed from the initial search records. The screening of the documents was discussed between these two authors, and any discrepancies were confirmed by another author.

For policy documents that met the criteria for this study, information was extracted, including: (1) background policy information such as the name of the policy document, year of issuance, issuing unit, and type of document; (2) information related to liver cancer prevention and control such as priority policies, government strategy, planning, program, projects that related to liver cancer prevention and control.

Regarding the time of release, the policy documents for liver cancer control in China were further grouped into five periods: the 10th Five-Year Plan (FYP) and earlier (1984–2005), the 11th FYP period (2006–2010), the 12th FYP n period (2011–2015), the 13th FYP period (2016–2020), and the 14th FYP period (2021-present). A FYP is a medium-term blueprint for the Chinese government to manage the economy and guide national development [32].

### Data analysis

**Fig. 1:**
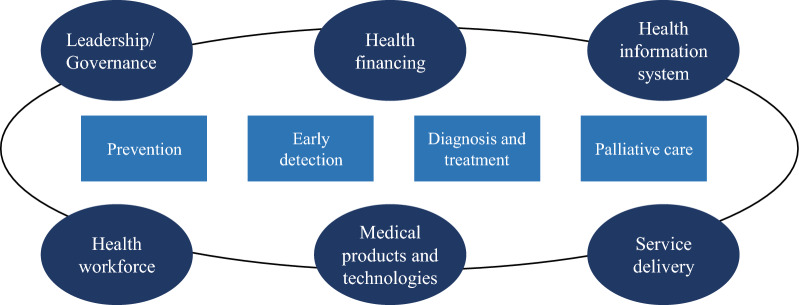
Combined theoretical framework

Two analytical frameworks were applied to the process of analysing policy information. The WHO Health System Six Building Blocks is widely used in health policy and health system research in a systemic thinking approach [33, 34]. The WHO Health System Six Building Blocks also can be used in conjunction with other policy analysis frameworks [35, 36]. The WHO Guidelines for Effective Cancer Control Programmes promote four modules of cancer control, including prevention, early detection, diagnosis and treatment, and palliative care [28–31]. Therefore, by combining the WHO Health System Six Building Blocks with WHO four cancer control modules in this study, the findings may offer a more intuitive presentation of the current imbalance in policies related to liver cancer control in China, as shown in Fig. 1. The indicators under the framework are shown in Appendix File 1.

## Results

### Characterstics of included policy documents

**Fig. 2:**
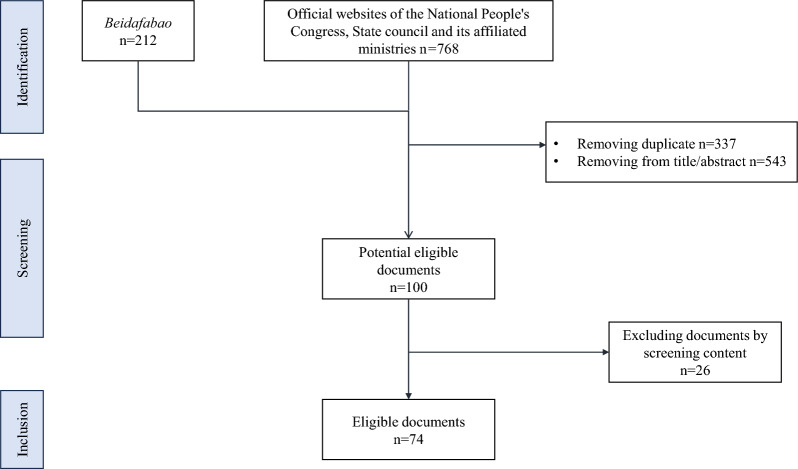
PRISMA flow diagram

A total of 980 records were retrieved from the policy search. After screening by title and context, 74 policies were included in this study [37–110]. The PRISMA flow diagram for the complete study selection process is presented in Fig. 2.

**Fig. 3:**
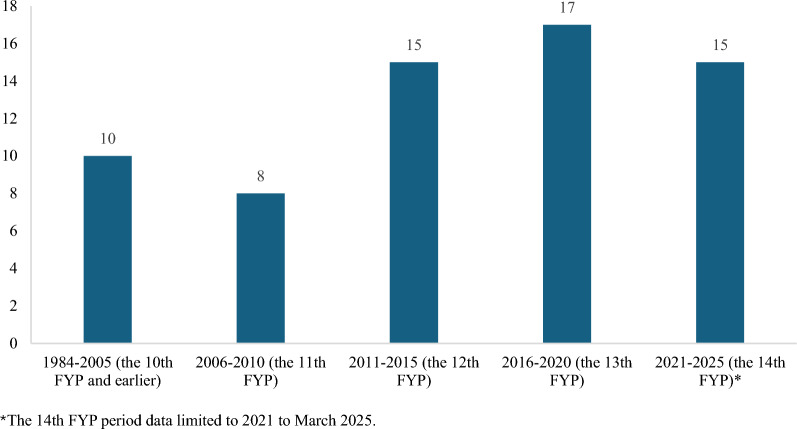
Trends of the release of policy documents on liver cancer in China

The number of liver cancer policy documents in China has gradually increased over the years, but the number of policies within each FYP is relatively stable (see Fig. 3). 52 (70.27%) documents included were issued by a single ministry or department. Furthermore, there is a collaborative effort involving multiple departments, such as the Ministry of Science and Technology's close cooperation aligns with China's emphasis on promoting the improvement of cancer diagnosis and treatment technologies, supporting the research and development of new anticancer drugs, and expediting the introduction of new drugs from overseas. This demonstrates that cancer control involves both health and non-health sectors. The specific information on included policies is shown in Appendix File 2.

**Fig. 4:**
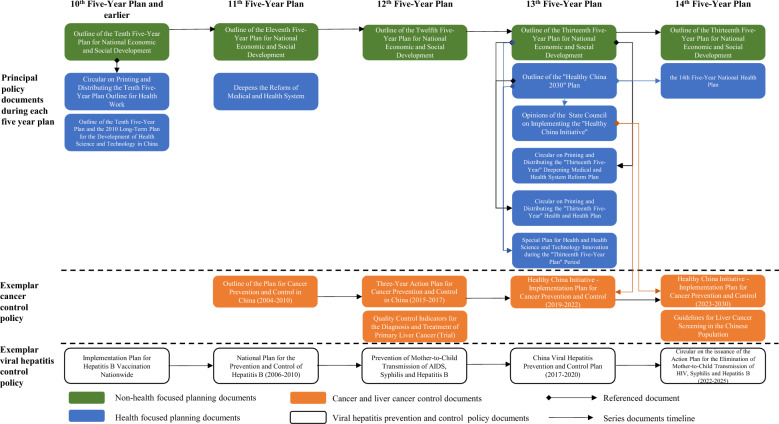
Leading polices and policy cross-referencing

Included policy documents can be divided into three levels: high-level and strategic planning policies (including medical and non-medical national planning policies), cancer control related policies (including documents related to cancer and liver cancer), and hepatitis prevention and control related policies (see Fig. 4). These policies show continuity over time. Among these policies, the most cited are high-level policies, especially the "Healthy China 2030" Plan (10 times), the Outline of the Thirteenth FYP for National Economic and Social Development (2016) (3 times), and Opinions of the State Council on Implementing the "Healthy China Initiative" (2 times).

### National liver cancer policy mapping

**Fig. 5:**
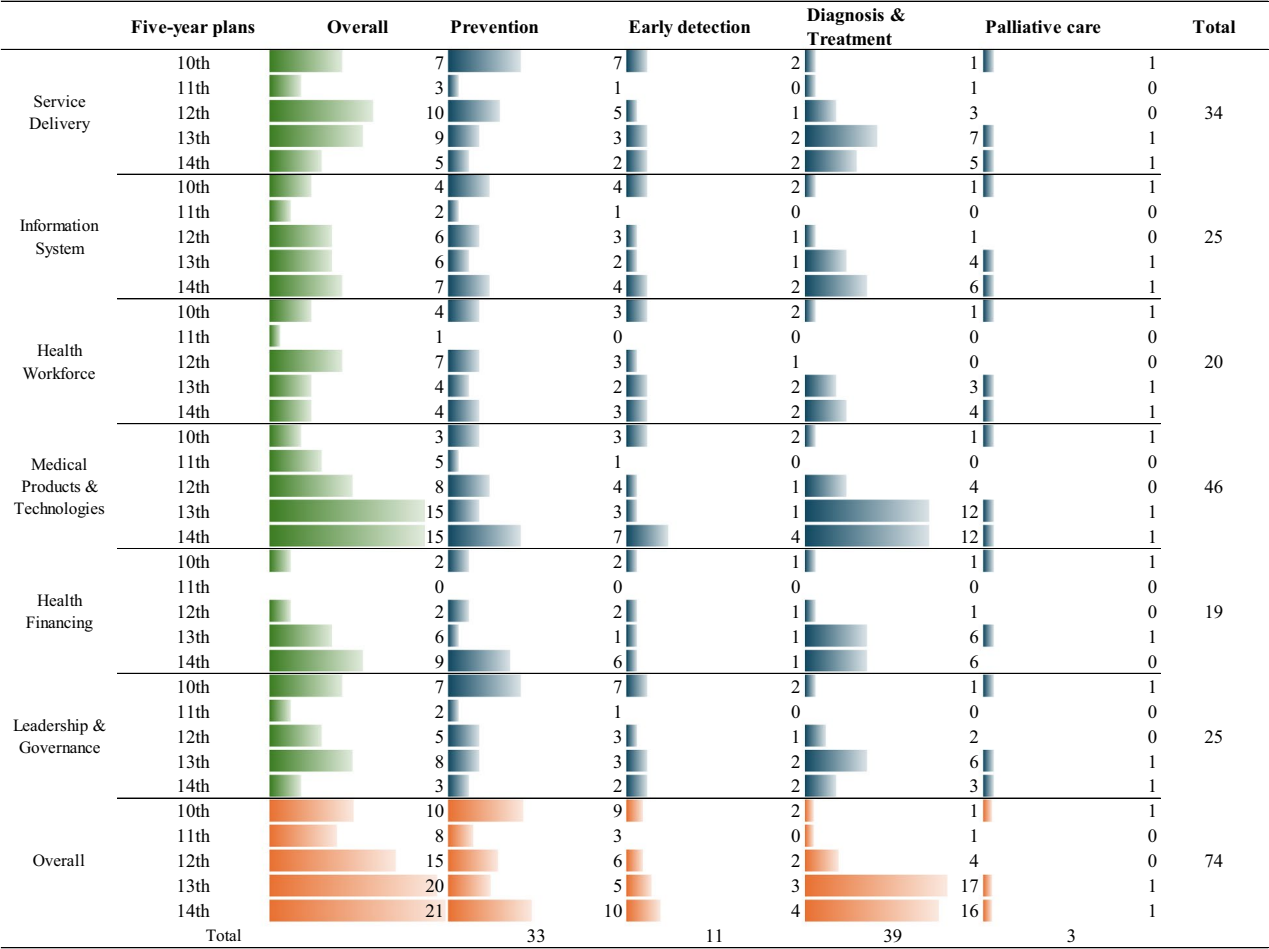
Number of policy documents mapped against the combined WHO six building blocks and WHO cancer control model

Under the WHO six building blocks framework, the included policy document covered each block (shown in Fig. 5), with *Medical products & Technology* and *Service delivery* being the most mentioned and showing a trend of gradually increasing in each FYP. The development of *Service delivery* was mentioned many times in 34 (45.95%) relevant policies, such as enhancing health workforce capabilities in diagnostics and treatment, and the establishment of clinical key cancer specialist diagnostic and treatment services. As for *Medical products & technology,* 46 (62.16%) relevant policies are issued to improve the availability of liver cancer and liver disease treatment drugs, and to improve liver cancer screening, diagnosis and treatment technologies. Notably, this study identified 19 (25.68%) policy documents that address *Health financing*, which is the least emphasized block based on WHO six building blocks. Detailed information about the analysis results was provided in Appendix File 3.

74 policy papers concerning liver cancer prevention and control were released during various periods. In the overall situation of the health system, the number of policies in the WHO six building blocks during the 13th FYP period was relatively high, showing a trend of increasing year by year in the number of policies.

Within the WHO four modules of cancer control, the most common policies focused on *Diagnosis & Treatment* of liver cancer (39, 52.70%). The second common policies focused on *Prevention* (33, 44.59%). There were fewer policies addressing *Early detection* (11,14.86%), and the number of policies about *Palliative care* was the lowest (3, 4.05%).

For *Prevention*, cancer prevention targets modifiable risk factors like tobacco, unhealthy diets, and infectious agents. Preventative measures offers the most cost-effective approach to long-term cancer control and often integrates with other chronic disease prevention [28]. In this study, 14 (18.92%) documents were related to the prevention of viral hepatitis. Since 1993, significant efforts have been made in the fields including hepatitis B vaccination and advancements in hepatitis B diagnosis and treatment, all of which have been implemented within the framework of the national immunization program.

For *Early detection*, relevant measures identify disease at initial stages when treatment is most effective, through patient symptom recognition or population screening of asymptomatic individuals [29]. The National Cancer Center organized a multidisciplinary team of experts to develop the “China guideline for liver cancer screening (2022, Beijing)” [98]. This guideline pointed out that screening is a systematic process that distinguishes apparently healthy but potentially diseased individuals from the truly disease-free population using cost-effective methods [98].

For *Diagnosis & Treatment*, relevant measures aims to cure disease, prolong life, and improve the quality of life following confirmed diagnosis of cancer [30]. For efficient and accurate diagnosis and treatment, the National Health Commission of the People's Republic of China published the clinical treatment guidelines and research practices for liver cancer [94, 108]. Besides, medication plays a crucial role in liver cancer treatment. To alleviate the financial burden associated with cancer medications, China conducted special negotiations in 2018 concerning anti-tumor drugs, as outlined in the “Circular of the National Bureau of Medical Security on the Inclusion of 17 Anti-cancer Drugs in Class B of the National Basic Medical Insurance, Work Injury Insurance and Maternity Insurance Drug Catalog”. This negotiation resulted in the inclusion of 17 drugs, with an average price reduction of 56.7% [81]. Before 2017, the medical insurance catalog did not include any oncology-targeted medications, but the latest 2024 catalog features more than 70 such drugs, improving accessibility and affordability for cancer patients [108].

*Palliative care* addresses symptom relief and psychosocial support needs of patients and families throughout the cancer journey, especially for advanced-stage patients with poor prognosis or terminal disease [31]. The policies mentioned promoting palliative care, standardizing palliative care plans, and improving the quality of life of patients with advanced cancer [44]. And it is also important to promote the formation of a continuous service model of diagnosis, treatment, rehabilitation, and long-term care, and provide rehabilitation and nursing services for patients with advanced cancer [73].

### Content analysis of initiatives in liver cancer control policy

The top ten most frequent policy keywords of included documents were hepatitis B, liver cancer, diagnostic and treatment approaches, early diagnosis and treatment, vaccination, viral hepatitis, prevention, screening, health insurance, and disease awareness.

We then aligned policy initiative examples with the four modules of cancer control, as detailed in Tables 2, 3, 4, and, 5, to illustrate different aspects of health system for liver cancer control in China. Liver cancer prevention policies primarily emphasized controlling viral hepatitis, particularly hepatitis B and hepatitis C.

**Table 2:** Prevention: identified primary strategies for liver cancer prevention and control with a focus on viral hepatitis prevention and control

WHO six building blocks	Examples of policy initiatives
Service delivery	Continuing promotion of viral hepatitis vaccination and integration of hepatitis B into the national immunization program [39, 47, 50], including enhancing hepatitis B vaccination and rolling out the hepatitis B vaccination initiative for newborns [69]; Strengthening hepatitis B vaccination among adults at high risk of hepatitis B virus infection [105]
Health information	Establish and improve a hepatitis C prevention and control information system based on the hepatitis C epidemic situation, population infection status and characteristics, disease outcomes, etc. [92, 99]
Health workforce	Enhancing the training of viral hepatitis healthcare professionals: for instance, between 2010 and 2012, 10,000 physicians specializing in relevant fields from 1,000 county hospitals in 15 high hepatitis B incidence provinces will receive training on standardized diagnosis and treatment of chronic hepatitis B [53]; Cultivating high-level scientific and technological talents and building an overall optimized health science and technology team [43]
Health financing	Multi-channel funding: promoting diverse and tailored health management service packages by social entities and investigating commercial health insurance as a financing or partnership avenue [95]
Medical products and technology	Promoting research and development of combined multivalent and genetically engineered vaccines [43]; Including more antiviral drugs for the treatment of hepatitis C that meet the selection principles into the national essential drug list [92]
Leadership and governance	Viral hepatitis prevention and control plans have been updated and released at different time stages, including the national hepatitis B prevention and control plan [99], the hepatitis C elimination plan [92], etc., and are continuously updated

**Table 3:** Early detection: identified prevention and control strategies for key populations with liver cancer

WHO six building blocks	Examples of policy initiatives
Service delivery	Strengthening public health services, screening and early detection of chronic diseases, and early diagnosis and treatment of key cancers in high-prevalence areas [72, 95]
Health information systems	Establishing an authoritative platform for spreading popular scientific information and coordinating professional organizations to gather and publish essential information and knowledge on cancer prevention and treatment. Carrying out initiatives for cancer informatization and enhancing the tumor registration system [84]
Health workforce	Strengthening the construction of cancer-related disciplines. Improve the structure of talent education and sound the multi-level training system for cancer prevention and treatment talents [84]
Health financing	Actively encouraging social capital to invest in cancer prevention and treatment, promoting the establishment of a diversified funding mechanism, and focusing the efforts of all parties to promote cancer prevention and treatment. [97]
Medical products and technology	It has strengthened the management of clinical application of antitumor drugs, guided medical institutions to do a good job in equipping and using negotiated anticancer drugs, and improved the guidelines for drug use [84]. Conduct research and development of early warning and early diagnosis reagents for hepatitis B-related liver cancer [76]. Release liver cancer screening guidelines 2022 [76, 99]
Leadership and governance	Continuing to promote early screening for key cancers, gradually expand the scope of screening, and strengthen the continuity of screening and early diagnosis and treatment [98]

**Table 4:** Diagnosis and Treatment: establishment of liver cancer diagnosis and treatment system based on standards for enhanced early diagnosis and treatment capabilities of healthcare institutions

WHO six building blocks	Examples of policy initiatives
Service delivery	Standardizing diagnostic and treatment practices. For example, the Quality Control Indicators for Standardized Diagnosis and Treatment of Liver Cancer in China (2022 Edition) were released to better promote the quality control of standardized diagnosis and treatment of liver cancer [100]
Health information systems	Establishing a comprehensive information management system. Enhance tumor registration and reporting, implement quality control standards, and improve reporting efficiency and quality. By 2030, ensure full coverage of tumor registration in all counties and districts, establish no less than 1,145 national tumor registries, and conduct high-precision tumor registration to collect crucial information [105]. Additionally, establish and refine the drug information traceability system [73]
Health workforce	Strengthening cancer disciplines and improving treatment in resource-limited areas by sharing expertise and technology. Innovate diagnosis and treatment models based on traditional Chinese medicine while training professionals in this field [105]
Health financing	Encourage social capital investment in cancer prevention and treatment, promote a diversified funding mechanism, and unite efforts to support and ensure progress in cancer care [105]
Medical products and technology	Promote research on hepatitis B and liver cancer treatments [76], advance cancer vaccine development, and streamline the approval of anti-tumor drugs. Facilitate the launch of new foreign drugs in China and establish import channels for urgently needed medications [105] Exploring and innovating cancer diagnosis and treatment modes in line with Chinese medicine theories, and cultivate cancer Chinese medicine, prevention and treatment professionals [84]
Leadership and governance	The policy focuses on standardizing liver cancer care in medical institutions to boost the five-year survival rate by 15% [94, 100]

**Table 5:** Palliative care: focusing on the health of people with advanced liver cancer

WHO six building blocks	Examples of policy initiatives
Service delivery	Promote palliative care, standardize palliative care plans, and improve the quality of life of patients with advanced cancer [44] Promote the formation of a continuous service model of diagnosis, treatment, rehabilitation, and long-term care, and provide rehabilitation and nursing services for patients with advanced cancer [73]
Health information systems	Medical institutions like the National Cancer Center and regional medical centers focusing on oncology need to enhance cooperation to improve the cancer prevention and control network [105]
Health workforce	Include cancer palliative treatment and pain relief programs in teaching. Encourage distance education [44]. Increase professional enrollment and training in rehabilitation and palliative care [105]
Health financing	Implement a long-term care insurance system [73]. Local governments must invest funds according to regulations, actively encourage private investment in cancer prevention and treatment, and coordinate efforts from all parties to guarantee progress in cancer prevention and treatment [105]
Medical products and technology	Utilize Chinese medicine and modern treatments to support patients in different stages, collaborating with Western medicine to manage symptoms [94]
Leadership and governance	Clarify medical service processes for acute and chronic diseases and establish a medical model of "treating minor illnesses in the community, addressing major illnesses in hospitals, and facilitating rehabilitation in the community" [73]

Early detection efforts aimed to identify individuals at high risk for liver cancer. Per the 2022 Chinese Liver Cancer Screening Guidelines, individuals at high risk include: (1) those with cirrhosis of any etiology (including alcoholic liver disease, and metabolic associated fatty liver disease), and (2) chronic HBV/HCV carriers aged ≥ 40 years [98]. To target high risk individuals with liver cancer, screening follows a two-stage protocol: (1) initial screening identifies high-risk individuals through HBsAg testing for viral hepatitis infection; (2) primary surveillance for high-risk cohorts employs semi-annual ultrasonography (US) combined with alpha-fetoprotein (AFP) testing. Abnormal test results will require further surveillance examinations. Indeterminate lesions will be required to undergo 3–6-month US/AFP re-evaluation, while high-risk lesions (e.g., AFP > 400 ng/mL) require immediate diagnostic imaging (Computed Tomography / Magnetic Resonance Imaging). Some blood serum markers, such as Protein Induced by Vitamin K Absence-II, circulating tumor DNA, micro-RNA are gradually being used as an adjunctive screening technique to help identify patients with hepatocellular carcinoma earlier [98].

Policies on diagnosis and treatment focused on enhancing the diagnostic and therapeutic capacities of medical institutions and establishing standards for hepatocellular carcinoma. Notably, there was less information regarding health financing in the documents examined. Additionally, palliative care had fewer associated policies, which emphasized patient support and pain relief for those with cancer without specific liver cancer.

### National programs of liver disease

In addition, this study examined liver disease and liver cancer prevention and control programs in China, as detailed in Table 6. A total of 8 national programs were searched [111–118]. Prior to 2019, the focus was predominantly on hepatitis prevention and control. In 2020, national-scale implementation began for programs targeting liver cancer prevention and control, including patient education and the treatment of high-risk groups. Recent years have witnessed an increased emphasis on national programs for the prevention and treatment of liver disease and liver cancer.

**Table 6:** National program for prevention and control of liver disease or liver cancer

Time	Program Name	Liver Cancer Control Content	Liver Cancer Prevention and Control Related Content
2008–2020	*Major Scientific and Technological Program for the Prevention and Control of Major Infectious Diseases, including AIDS and Viral Hepatitis* [114]	Prevention	Viral hepatitis vaccination to reduce hepatitis b prevalence rates
2008–2020	*Major Scientific and Technological Program for the Development of New Drugs* [117]	Diagnosis and treatment	Promoting the development of drug discovery systems and enhancing the accessibility and availability of oncology medications
2016	*“Demonstration Center for the Prevention and Treatment of Hepatitis B in Pregnancy”* [115]	Prevention	Establishing hepatitis B control demonstration bases to reduce the risk of mother-to-child transmission of hepatitis B
2017	*China Quality Liver Disease Medical Resources County Support Initiative* [118]	Prevention, Diagnosis and treatment	Enhancing Liver Disease Diagnosis and Treatment Standards in Medical Institutions
2017–2019	*"Fight Hepatitis B: Green Home—Health Education Program for Chronic Hepatitis B Patients"* [111]	Prevention	Guide hepatitis B patients and their families to understand and adhere to standardized treatment to improve outcomes and quality of life
2018	*China's Clinical Cure Project for Chronic Hepatitis B (Everest project)* [112]	Prevention	Enhancing liver disease diagnosis and treatment standards in medical institutions
2022	*China’s Study on Reducing the Incidence of Liver Cancer in Hepatitis B Patients (Oasis project)* [113]	Prevention, Diagnosis and treatment	Improving the treatment of hepatitis b-associated hepatocellular carcinoma
2021	*Early Screening for People at Risk of Liver Cancer- the Ganlin Programme* [116]	Prevention	Increasing the level of cancer awareness, consultation rates, and early detection rates of liver cancer among high-risk groups in China

In terms of liver cancer prevention, some projects have done a lot of work on the prevention of viral hepatitis. For example, the *Green Home Project* aims to raise hepatitis B awareness among patients and families through publicity. From 2017 to 2019, 900 education sessions reached more than 30,000 patients. Their WeChat account has over 34,000 followers and 196 posts with nearly 2 million reads [111].

The *Everest Project* is a multicenter research project on the cure of chronic hepatitis B in China. Its research results confirmed the clinical cure of pegylated interferon α (INF α) in nucleoside analog suppressed chronic hepatitis B [112]. Besides, the *Oasis Project*, one of Asia's largest hepatitis B cohorts, has enrolled 32,071 patients from 295 hospitals since its launch in 2020. Interim results suggest that for patients with higher hepatitis B surface antigen levels, extending IFNα treatment time or using intermittent pegylated interferon-based treatment may be effective for achieving a functional cure [113]. The *Major Scientific and Technological Program for the Prevention and Control of Major Infectious Diseases, including AIDS and Viral Hepatitis* has better controlled new hepatitis B infections and significantly reduced the hepatitis B infection rate in children. This program achieved reducing the mortality rate of chronic severe hepatitis from 84.6% to 56.6% [114]. In 2012, China has achieved the World Health Organization's goal for chronic hepatitis B infection in children under five years ahead of schedule, with a rate of less than 1%, and by 2014, the rate had dropped to 0.32%, achieving mother-to-child transmission interruption with a success rate of over 99% [115].

For early detection of liver cancer, the *Ganlin Project* aims to train over 1,000 liver disease specialists in more than 100 hospitals located in areas with a high incidence of liver cancer nationwide. The project also aims to conduct early screening for 5,000 individuals who are at a high risk of developing liver cancer [116].

For treatment of liver cancer, the *Major Scientific and Technological Program for the Development of New Drugs,* Danoprevir sodium, a drug independently developed for viral hepatitis C, passed the priority review process and was approved for marketing faster [117]. The National Health Commission-managed Chines Foundation for Hepatitis Prevention and Control implements nationwide initiatives to standardize regional liver disease care. Through a hub-and-spoke model, central hospitals enhance district/county capacity via expert consultations, patient education, and infrastructure support, thereby elevating primary-level diagnostic and treatment quality for liver cancer and other liver diseases [118].

## Discussion

To our knowledge, this study is the first to provide an overview of national-level policies for controlling liver cancer in China. Over the past 40 years, 74 documents on liver cancer policy have been published. The focus has been on prevention and diagnosis and treatment, while there is a lack of documents mentioned about early detection and palliative care for liver cancer control. From the perspectives of health system responses, service delivery, medical products and technologies are the two major areas mentioned in most policy documents.

Viral hepatitis prevention forms the cornerstone of China's liver cancer prevention. Since 1984, China has consistently prioritized the control of viral hepatitis and has incorporated the prevention of bacterial and viral infections linked to cancer development into its national cancer program [105]. The study results show that China has made significant progress in preventing hepatitis B in mothers and infants. Through sustained efforts over two decades, China has achieved 99.6% of a three-dose hepatitis B vaccine coverage rate, with birth-dose vaccination coverage reaching 95.6%, which is significantly lower than the global average [119]. Correspondingly, the mother-to-child transmission rate of hepatitis B virus (HBV) has declined to 0.23% [119]. In the future, China plans to expand its efforts to prevent hepatitis B in more people and continue promoting hepatitis C prevention. However, China still bears a significant portion of the global burden of HBV [120], with nearly one-third of the global infection cases and over 300,000 people dying from HBV-related illnesses each year [121]. In light of the targets of the *Global Health Sector Strategies on HIV, viral hepatitis and sexually transmitted infections (STIs) 2022–2030 (GHSS)* [122], China has planned to further intensify its efforts in addressing viral hepatitis, especially in improving diagnostic, linkage-to-care, and treatment coverages [123, 124].

In the *Early detection* of liver cancer, screening has been proved to be the most crucial and effective module [125]. Previous experts’ consensus and professional guidelines have established a foundation for liver cancer screening in China [126, 127]. However, the current population coverage rate for liver cancer screening in China remains at only 0.09% [128], indicating that further efforts are needed to promote and expand screening programs. Except for screening, surveillance policy for liver cancer in precarious populations is blank in current documents.

Especially, the rising non-alcoholic fatty liver disease (NAFLD)/non-alcoholic steatohepatitis (NASH) related liver cancer in China indicates a new demand for the early detection of liver cancer. It is estimated that about 38% NAFLD or NASH patients will directly progress to liver cancer without liver cirrhosis stage [129]. Global guidelines recommend risk-stratified liver cancer surveillance for NAFLD/NASH patients, integrating Fibrosis-4 (FIB-4) scores, liver stiffness measurement via transient elastography, and ultrasound (US) [130–132]. The 2020 Chinese Preventive Medicine Association guidelines stratify non-cirrhotic NASH/NAFLD individuals into low-risk (liver cancer incidence < 1%) and medium-risk (1–3%) cohorts. Biennial US-AFP surveillance is recommended for low-risk individuals, while annual monitoring is advised for medium-risk groups [133]. However, evidence-based preventive strategies for non-cirrhotic NASH/NAFLD patients remain insufficient, with critical gaps including lacking validated biomarkers for early detection [134, 135], limited methods or tools for recognition of at-risk patients [136], lack of consensus regarding the value of surveillance in non-cirrhotic NAFLD [137], and insufficient long-term cost-effectiveness data [138]. Besides, suboptimal adherence to screening strategies by patients and healthcare providers further impedes prevention [139, 140].

As for the *Diagnosis &Treatment* of liver cancer, there are specialized guidelines with solid evidence of novel randomized clinical trials and observational findings [94]. There is a need to integrate and optimize advanced treatment strategies from domestic and international sources through optimized study designs such as prospective and multicenter trials, especially for combination therapies [141, 142]. Considering the aggressive nature of HBV-associated hepatocellular carcinoma (HCC), real-life practices in China have adopted multimodal treatments, such as the addition of immunotherapy-based systemic treatment into local therapies [143]. Besides, more than 20 phase III trials are currently underway to determine the effectiveness of immunotherapy-based therapies in the various stages of HCC [144].

Notably, the policies also focus on promoting the role of traditional Chinese medicine in cancer treatment in China [108]. Traditional Chinese medicine offers new options and challenges for liver cancer management. The anti-tumor efficacy of traditional Chinese medicine has been widely recognized [145]. There are a few approved Chinese patent medicines that expand the therapeutic options for primary liver cancer, including Huaier granules, Ganfule granules, Fufang Banmao capsules, Jinlong capsules, Brucea javanica oil emulsions, and compound kushen injections [106]. In addition to this, Chinese herbal medicine as an adjuvant treatment for liver cancer has also demonstrated better clinical results, with a real-world cohort study of 2,067 patients finding that it reduces mortality and prolongs survival time [146]. On top of that, traditional Chinese medicine and natural active products such as evodiamine, croton, solanine, vinblastine, etc., have anti-tumor activity in treating liver cancer [147]. Future research should address potential toxicity and low bioavailability of Chinese medicine in treating liver cancer and conduct large-sample, long-term clinical trials to ensure effectiveness and safety [148].

This study found that national policies for liver cancer control focused on *Medical products & Technologies* and *Service delivery* under the analysis of the WHO Health System six building blocks framework. For *Medical Products & Technologies,* the development of drugs for liver cancer or hepatocellular carcinoma, as one of the key cancers in China, is the focus of a major national new drug development project [115]. In recent years, China has approved several first-in-class liver cancer treatment drugs, including Sintilimab Injection, Camrelizumab Injection, Apatinib Mesylate Tablets, Dominafinone Tosilate Tablets, and Tislelizumab Injection [109]. Fast-track pathway for cancer and other major diseases have expedited access to foreign therapies for patients.

However, the supply of antiviral hepatitis drugs was not ideal. A cross-sectional study showed that the availability of anti-hepatitis B drugs in Jiangsu Province, China was less than 30%, the affordability rate for urban residents was 23%, and the affordability rate for rural residents was 0% [149]. In addition, real-world studies have shown that new Hepatitis C Virus treatment options were not cost-effective [150]. For liver cancer drug therapy, cost-effectiveness research is still a hot topic, especially on hepatocellular carcinoma [151, 152].

Besides, for *Service delivery*, the Chinese government has been continuously working to improve the professional service capacity of medical institutions. In China, the accessibility of liver cancer screening services is not good. In 2019, only about 0.09% of people aged 35–74 received screening through the national liver cancer screening program [145]. China still needs to promote the further implementation of services for liver cancer.

Regarding *Health financing*, which has the smallest number of related policies, we need to recognize the gap between our current health spending and that of developed countries. China spent $1.3 trillion on health, accounting for 7.2% of GDP [153]. Among them, government health expenditure accounted for 26.7%; social health expenditure accounted for 46.0%; and out-of-pocket health expenditure accounted for 27.3% [153]. In terms of cancer control, priority in the financing is given to cancer screening. The government allocates 280 million yuan annually to support cancer screening in high-risk areas [154]. Healthcare spending should be effectively utilized, and health policies should be improved to enhance investment in health technology [154, 155].

Evaluation of the national cancer control plan and its implementing programs will help improve policy effectiveness. In 2002, the WHO released national cancer control programs, which provided a framework for developing cancer control policies [156]. Till now, nearly 133 countries have developed NCCPs. Studies suggest that liver cancer is the second type of cancer anticipated to be eliminated following cervical cancer [14]. Consequently, there is a pressing need for the development of targeted liver cancer prevention and control strategy at the national level in China to reduce the disease burden.

Furthermore, various tools could be integrated to support development and evaluation of a national liver cancer prevention and control policy. Examples for reference include the National Cancer Control Programs Core Capacity Self-Assessment Tool of WHO [157], the Cancer Plan Development and Implementation Assessment Tool of ICCP [158], and the European Guide for Quality National Cancer Control Programs from European Partnership Action Against Cancer [159]. These tools would assist in timely assessing the effectiveness of national liver cancer programs. Additionally, incorporating system thinking methodologies, such as the system dynamics model [160], could help identify potential influencing factors during the implementation of liver cancer interventions. This approach would allow for dynamic adjustments to policies based on evolving insights and outcomes. These frameworks and tools could support the development of a national liver cancer strategy in China.

### Limitations

This study has several limitations. First, the policy documents included in this study were as of 31 March 2025, and the analysis of the documents for the 14th FYP period is incomplete. Second, data were collected from publicly searchable databases and media sources, so unpublished policies may not have been retrieved. Third, this research policy search only included documents related to “liver”in the search policy documents and did not include national non-communicable diseases and other policies, which was a relatively macro-level analysis. Fourth, this study was limited to policy text analysis and did not quantitatively evaluate the pre- and post-implementation effects of liver cancer policies. Future research and exploration of liver cancer control policies in China should focus on ongoing tracking of policy developments, comparative analysis of implementation outcomes, and the heterogeneity of regional effects.

## Conclusions

This study provides an in-depth analysis of China's national policies for the prevention and control of liver cancer, highlighting a 40-year effort with 74 relevant policy documents focusing more on prevention, diagnosis, and treatment through strengthening service delivery and medical products but less on detection and palliative care. Future efforts should prioritize the development of a holistic national liver cancer strategy based on a dynamic evaluation of the health system.

## Supplementary Information

Additional file 1.

Additional file 2.

Additional file 3.

## Data Availability

The data used and/or analysed during the current study is included within the article and its additional files.

## Abbreviations

AIDS: Acquired immune deficiency syndrome
FYP: Five-year plan
GDP: Gross domestic product
GHSS: Global health sector strategies
HBV: Hepatitis B virus
HCC: Hepatocellular carcinoma
HCV: Hepatitis C virus
HIV: Human immunodeficiency virus
ICCP: The International cancer control partnership
INF α: Interferon α
NAFLD: Non-alcoholic fatty liver disease
NASH: Non-alcoholic steatohepatitis
NCCP: National cancer control program
PRC: The People's Republic of China
STIs: Sexually transmitted infections
WHO: The World Health Organization
