# Supplementary material for: Critical analysis of the liver cancer policies and programs in China: implications for international liver cancer control
Source: Glob Health Res Policy. 2025 Oct 2;10:50. doi: 10.1186/s41256-025-00450-w (PMC12490094; doi:10.1186/s41256-025-00450-w)
Supplement: Supplementary file 1 — Additional file 1. [file 41256_2025_450_MOESM1_ESM.docx]

Supplementary File 1. Table. WHO Health System Six Building Blocks and core indicators^1^

| **Building blocks** | **Indicators** |
| --- | --- |
| 1. Health service delivery | - Number and distribution of health facilities per 10 000 population - Number and distribution of inpatient beds per 10 000 population - Number of outpatient department visits per 10 000 population per year - General service readiness score for health facilities - Proportion of health facilities offering specific services - Number and distribution of health facilities offering specific services per 10 000 population - Specific-services readiness score for health facilities |
| 2. Health Workforce | - Annual number of graduates of health professions educational institutions per 100,000 population, by level and field of education - Distribution of health workers by occupation/specialization, region, place of work and sex - Number of health workers per 10,000 population |
| 3. Health Information Systems | - Health information system performance index: health surveys; birth and death registration; census; health facility reporting; health system resource tracking; capacity for analysis, synthesis and validation for health data |
| 4. Medical Products and Technologies | - Median consumer price ratio of 14 selected essential medicines in public and private health facilities - Average availability of 14 selected essential medicines in public and private health facilities |
| 5. Health Systems Financing | - Total expenditure on health - General government expenditure on health as a proportion of general government expenditure (GGHE/GGE) - The ratio of household out-of-pocket payments for health to total expenditure on health |
| 6. Leadership and Governance | - Existence of an up-to-date national health strategy linked to national needs and priorities - Existence and year of last update of a published national medicines policy - Existence of policies on medicines procurement that specify the most cost-effective medicines in the right quantities; open, competitive bidding of suppliers for quality products - Tuberculosis—existence of a national strategic plan for tuberculosis that reflects the six principal components of the Stop-TB strategy as outlined in the Global Plan to Stop TB 2006–2015 - Malaria—existence of a national malaria strategy or policy that includes drug efficacy monitoring, vector control and insecticide resistance monitoring - HIV/AIDS—completion of the UNGASS National Composite Policy Index questionnaire for HIV/AIDS - Maternal health—existence of a comprehensive reproductive health policy consistent with the ICPD action plan - Child health—existence of an updated comprehensive, multiyear plan for childhood immunization - Existence of key health sector documents that are disseminated regularly (such as budget documents, annual performance reviews and health indicators) - Existence of mechanisms, such as surveys, for obtaining opportune client input on appropriate, timely and effective access to health services |

Abbreviations: TB, Tuberculosis; HIV, Human immunodeficiency virus; AIDS, Acquired Immune Deficiency Syndrome; UNGASS, United Nations Children’s Education Fund; ICPD, the International Conference on Population and Development.

References:

1. World Health Organization. Monitoring the building blocks of health systems: a handbook of indicators and their measurement strategies. Geneva, Switzerland: WHO Document Production Services. 2010.
