# Supplementary material for: Critical analysis of the liver cancer policies and programs in China: implications for international liver cancer control
Source: Glob Health Res Policy. 2025 Oct 2;10:50. doi: 10.1186/s41256-025-00450-w (PMC12490094; doi:10.1186/s41256-025-00450-w)
Supplement: Supplementary file 2 — Additional file 2. [file 41256_2025_450_MOESM2_ESM.docx]

Supplementary File 2.

Table. Basic information on included national-level policy documents related to liver cancer in China

| **Reference no** | **Issued Time** | **Policy Name** | **Disease** | **Type of Control** | **Issuing Department** | **Policy types** |
| --- | --- | --- | --- | --- | --- | --- |
| 1 | 1984.04.18 | Plan for the Prevention and Control of Viral Hepatitis | Hepatitis | Prevention | Ministry of Health of PRC. | Implementation document |
| 2 | 1987.09.14 | Trial Measures for Immunization of Hepatitis B Blood-Derived Vaccine Nationwide | Hepatitis B | Prevention | Ministry of Health of PRC. | Implementation document |
| 3 | 1991.10.25 | National Implementation Plan for Hepatitis B Vaccine Immunization | Hepatitis B | Prevention | Ministry of Health of PRC. | Implementation document |
| 4 | 1993.08.16 | Circular on Further Strengthening the Current Prevention and Control of Hepatitis | Hepatitis | Prevention | Ministry of Health of PRC, Ministry of Finance of PRC. | Implementation document |
| 5 | 2001.07.23 | Circular on Printing and Distributing the Tenth Five-Year Plan Outline for Health Work | General Liver Disease | Prevention | Ministry of Health of PRC. | Official document |
| 6 | 2001.12.05 | Circular on Including Hepatitis B Vaccine in Children's Immunization Program | Hepatitis B | Prevention | Ministry of Health of PRC. | Official document |
| 7 | 2001.12.13 | Outline of the Tenth Five-Year Plan and the 2010 Long-Term Plan for the Development of Health Science and Technology in China | General Liver Disease - General Tumor, Viral Hepatitis | Prevention | Ministry of Health of PRC. | Official document |
| 8 | 2004.02.25 | Outline of the Plan for Cancer Prevention and Control in China (2004-2010) | Liver Cancer | Prevention, Early Detection, Surveillance, Diagnosis and Treatment, Palliative Care | Ministry of Health of PRC. | Official document |
| 9 | 2005.03.21 | Guiding Opinions on the Training Outline for Rural Health Workers | General Liver Disease | Other - Health Services - Talent | Ministry of Health of PRC. | Official document |
| 10 | 2005.12.29 | Outline of the National Medium- and Long-Term Science and Technology Development Plan (2006-2020) | General Liver Disease - Hepatitis B; General Tumor | Prevention, Early Detection | Ministry of Health of PRC. | Official document |
| 11 | 2006.01.28 | National Plan for the Prevention and Control of Hepatitis B (2006-2010) | Hepatitis B | Prevention | Ministry of Health of PRC. | Official document |
| 12 | 2006.09.02 | Key Points of Knowledge for Publicity and Education on Prevention and Control of Hepatitis B | Hepatitis B | Prevention | The State Council. | Implementation document |
| 13 | 2007.01.23 | Management Measures for Early Diagnosis and Treatment of Cancer Projects (Trial) | Liver Cancer | Diagnosis and Treatment | Ministry of Health of PRC. | Implementation document |
| 14 | 2008.05.02 | Circular on Printing and Distributing the Implementation Plan for Expanding the National Immunization Program | Hepatitis; Hepatitis B and C | Prevention | Ministry of Health of PRC. | Official document |
| 15 | 2008.12.01 | Circular on Printing and Distributing the Basic Responsibilities of Disease Prevention and Control Institutions at All Levels and the Performance Evaluation Standards for Disease Prevention and Control Work | General Liver Disease | Other - Health Services | Ministry of Health of PRC. | Implementation document |
| 16 | 2008.12.15 | Circular on Establishing the National Traditional Chinese Medicine Clinical Research Base TCM Prevention and Treatment of Liver Disease Clinical Research Alliance | General Liver Disease | Other - Health Services | General Office of Ministry of Health of PRC. | Implementation document |
| 17 | 2010.07.16 | Circular on Carrying out the Standardized Diagnosis and Treatment Training Program for Chronic Hepatitis B in County-Level Hospitals | Hepatitis B | Other - Health Services - Talent | General Office of Ministry of Health of PRC. | Implementation document |
| 18 | 2010.12.14 | Guidelines for the Standardized Diagnosis and Treatment of Common Tumors in Municipal and County-Level Hospitals (Trial) | General Liver Disease - General Tumor | Other - Health Services | National People's Congress. | Implementation document |
| 19 | 2011.02.24 | Circular on Printing and Distributing the Implementation Plan for the Prevention of Mother-to-Child Transmission of AIDS, Syphilis and Hepatitis B | Hepatitis B | Prevention | General Office of Ministry of Health of PRC. | Implementation document |
| 20 | 2011.03.16 | Outline of the Twelfth Five-Year Plan for National Economic and Social Development of the People's Republic of China | General Liver Disease - General Tumor | Surveillance, Diagnosis and Treatment | Ministry of Health of PRC. | Official document |
| 21 | 2011.05.24 | National Basic Public Health Service Specification (2011 Edition) | General Liver Disease | Other - Health Services | Ministry of Health of PRC. | Implementation document |
| 22 | 2011.07.12 | Circular on Carrying out the 2011 World Hepatitis Day Publicity Activities | Hepatitis | Prevention | General Office of Ministry of Health of PRC. | Implementation document |
| 23 | 2012.02.22 | Guidelines for the Construction and Management of Liver Disease Departments in Traditional Chinese Medicine Hospitals and Other Five Departments (Trial) | General Liver Disease | Other - Health Services | Ministry of Health of PRC. | Implementation document |
| 24 | 2012.07.06 | Circular on Organizing and Carrying out the 2012 World Hepatitis Day Publicity Activities | Hepatitis | Prevention | The State Council of PRC. | Implementation document |
| 25 | 2012.10.08 | Circular on Printing and Distributing the Twelfth Five-Year Plan for the Development of Health Undertakings | General Liver Disease - General Tumor | Surveillance, Diagnosis and Treatment | National Administration of Traditional Chinese Medicine, Ministry of health, Ministry of Education of PRC. | Official document |
| 26 | 2012.10.26 | Management Measures for Urban Cancer Early Diagnosis and Early Treatment Projects | General Liver Disease - General Tumor | Prevention, Early Detection | Ministry of Health of PRC. | Implementation document |
| 27 | 2012.11.08 | Evaluation Standards for the Construction of National Clinical Key Specialties (Trial) | General Liver Disease | Other - Health Services | Ministry of Health of PRC, Ministry of Finance of PRC, Ministry of Commerce of PRC, National Administration of Traditional Chinese Medicine. | Implementation document |
| 28 | 2012.12.14 | Quality Control Indicators for the Diagnosis and Treatment of Primary Liver Cancer (Trial) | Liver Cancer | Diagnosis and Treatment | National Administration of Traditional Chinese Medicine. | Implementation document |
| 29 | 2013.02.17 | Standardized Training Standards for General Practitioners of Traditional Chinese Medicine (Trial) | General Liver Disease | Other - Health Services - Talent | National Administration of Traditional Chinese Medicine, National Health and Family Planning Commission of PRC, Ministry of Education of PRC. | Implementation document |
| 30 | 2014.08.07 | Circular on Printing and Distributing the Work Plan for Comprehensively Improving the Comprehensive Capability of County-Level Hospitals | General Liver Disease | Other - Health Services | Ministry of Health of PRC. | Official document |
| 31 | 2014.12.11 | Implementation Measures for the Standardized Training of Traditional Chinese Medicine Residents (Trial) | General Liver Disease | Other - Health Services - Talent | National Health and Family Planning Commission of PRC, National Administration of Traditional Chinese Medicine. | Implementation document |
| 32 | 2015.06.16 | Circular on Fully Implementing the Prevention of Mother-to-Child Transmission of AIDS, Syphilis and Hepatitis B | Hepatitis B | Prevention | National Health and Family Planning Commission of PRC, National Development and Reform Commission of PRC, Ministry of Education of PRC. | Implementation document |
| 33 | 2015.09.09 | Three-Year Action Plan for Cancer Prevention and Control in China (2015-2017) | Liver Cancer, Hepatitis B | Prevention, Early Detection, Diagnosis and Treatment | The Communist Party of China (CPC) Central Committee, the State Council. | Official document |
| 34 | 2016.02.16 | Circular on Printing and Distributing the Training Outline for Newly Recruited Nurses (Trial) | General Liver Disease | Other - Health Services - Talent | National Health and Family Planning Commission of PRC, National Development and Reform Commission of PRC, Ministry of Education of PRC, Ministry of Science and Technology of PRC, Ministry of Industry and Information Technology of PRC, Ministry of Public Security of PRC, Ministry of Finance of PRC, Ministry of Human Resources and Social Security of PRC, Ministry of Housing and Urban-Rural Development of PRC, China Food and Drug Administration, National Administration of Traditional Chinese Medicine. | Implementation document |
| 35 | 2016.03.17 | Outline of the Thirteenth Five-Year Plan for National Economic and Social Development of the People's Republic of China | General Liver Disease - General Tumor | Surveillance, Diagnosis and Treatment | General Office of National Health and Family Planning Commission of PRC. | Official document |
| 36 | 2016.10.25 | Outline of the "Healthy China 2030" Plan | General Liver Disease - General Tumor | Prevention, Diagnosis and Treatment, Early Detection, Surveillance | The State Council. | Official document |
| 37 | 2016.12.27 | Circular on Printing and Distributing the "Thirteenth Five-Year" Deepening Medical and Health System Reform Plan | General Liver Disease - General Tumor | Palliative Care, Diagnosis and Treatment | The State Council. | Official document |
| 38 | 2016.12.27 | Circular on Printing and Distributing the "Thirteenth Five-Year" Health and Health Plan | General Liver Disease - General Tumor | Surveillance, Diagnosis and Treatment | National People's Congress. | Official document |
| 39 | 2017.01.11 | Special Plan for Health and Health Science and Technology Innovation during the "Thirteenth Five-Year Plan" Period | Liver Cancer | Prevention | National Health Commission of PRC, | Official document |
| 40 | 2017.10.17 | China Viral Hepatitis Prevention and Control Plan (2017-2020) | Hepatitis | Prevention | The State Council. | Official document |
| 41 | 2018.03.28 | Circular on Publishing 7 Health Industry Standards Including "Diagnosis of Hepatitis C" | Hepatitis C | Diagnosis and Treatment | National Healthcare Security Administration. | Implementation document |
| 42 | 2018.07.17 | Circular on Carrying out Provincial-Level Centralized Procurement of Anti-Cancer Drugs | Liver Cancer | Diagnosis and Treatment | National Health Commission of PRC, National Administration of Traditional Chinese Medicine. | Implementation document |
| 43 | 2018.08.20 | Circular on Printing and Distributing the Key Tasks of Deepening Medical and Health System Reform in the Second Half of 2018 | General Liver Disease - General Tumor | Diagnosis and Treatment | National Healthcare Security Administration. | Implementation document |
| 44 | 2018.09.21 | the Clinical Application Guidelines for New Antineoplastic Drugs (2018 Edition) | Liver Cancer | Diagnosis and Treatment | National Health Commission of PRC | Implementation document |
| 45 | 2018.09.30 | Circular from the National Medical Security Administration on Including 17 Anti-Cancer Drugs in the Category B List of National Basic Medical Insurance, Work Injury Insurance and Maternity Insurance Drug Catalogue | Liver Cancer | Diagnosis and Treatment | General Office of the State Council. | Implementation document |
| 46 | 2018.10.15 | 2018 Edition of the National Essential Drug List | General Liver Disease | Diagnosis and Treatment | National Health Commission of PRC, National Develoment and Reform Commission of PRC, Ministry of Education of PRC, Ministry of Science and Technology of PRC, Ministry of Ecology and Environment of PRC, National Healthcare Security Administration, National Administration of Traditional Chinese Medicine, National Medical Products Administration, Poverty Relief Office of the State Council. | Implementation document |
| 47 | 2019.07.15 | Opinions of the The State Council on Implementing the "Healthy China Initiative" | General Liver Disease - General Tumor | Prevention, Diagnosis and Treatment, Early Detection, Surveillance | National Healthcare Security Administration. | Official document |
| 48 | 2019.09.20 | Healthy China Initiative - Implementation Plan for Cancer Prevention and Control (2019-2022) | General Liver Disease - General Tumor | Prevention, Diagnosis and Treatment, Early Detection, Surveillance | The State Council. | Implementation document |
| 49 | 2019.12.20 | the Clinical Application Guidelines for New Antineoplastic Drugs (2019 Edition) | Liver Cancer | Diagnosis and Treatment | National Health Commission of PRC | Implementation document |
| 50 | 2020.06.18 | Circular from the Office of the National Medical Security Administration on Printing and Distributing the Detailed Grouping Scheme for Disease Diagnosis Related Groups (CHS-DRG) (Version 1.0) | General Liver Disease | Diagnosis and Treatment | National Medical Products Administration, National Health Commission of PRC. | Implementation document |
| 51 | 2020.07.12 | 2020 Edition of the "Pharmacopoeia of the People's Republic of China" | General Liver Disease | Diagnosis and Treatment | General Office of National Health Commission of PRC, General Office of Ministry of Science and Technology of PRC, General Office of Ministry of Industry and Information Technology of PRC, General Office of Ministry of Public Security of PRC, General Office of Ministry of Civil Affairs of PRC, General Office of Ministry of Justice of PRC, General Office of Ministry of Finance of PRC, Office of National Healthcare Security Administration, National Medical Products Administration. | Implementation document |
| 52 | 2020.11.20 | Circular on the Issuance of the National Health Insurance Fractional Payment by Disease (DIP) Technical Specifications and the DIP Disease Catalog Library (Version 1.0) | General Liver Cancer | Diagnosis and Treatment | General Office of National Health Commission of PRC. | Implementation document |
| 53 | 2020.12.30 | the Clinical Application Guidelines for New Antineoplastic Drugs (2020 Edition) | Liver Cancer | Diagnosis and Treatment | National Health Commission of PRC | Implementation document |
| 54 | 2021.04.12 | Circular on Accelerating Cross-Provincial Direct Settlement of Outpatient Expenses | General Liver Cancer | Diagnosis and Treatment | Office of National Healthcare Security Administration. | Implementation document |
| 55 | 2021.09.14 | Circular on the Pilot Work of Cross-Provincial Direct Settlement of Treatment Costs Related to Outpatient Chronic and Special Diseases | General Liver Cancer | Diagnosis and Treatment | National Healthcare Security Administration, Ministry of Finance of PRC. | Implementation document |
| 56 | 2021.09.15 | Circular on Printing and Distributing the Action Plan for Eliminating Public Health Hazards of Hepatitis C (2021-2030) | Hepatitis C | Prevention | Office of National Healthcare Security Administration, General Office of Ministry of Finance of PRC. | Implementation document |
| 57 | 2021.11.03 | Circular on the Issuance of Criteria for the Establishment of National Cancer Medical Centers | General Liver Cancer | Prevention, Diagnosis and Treatment, Early Detection, Surveillance, Palliative Care | General Office of the State Council. | Implementation document |
| 58 | 2022.01.21 | Standard for diagnosis and treatment of primary liver cancer(2022 edition) | Primary Liver Cancer | Diagnosis and Treatment | National Health Commission of PRC. | Implementation document |
| 59 | 2022.04.27 | the 14th Five-Year National Health Plan | Hepatitis C | Prevention, Diagnosis and Treatment, Early Detection, Surveillance | General Office of National Health Commission of PRC. | Official document |
| 60 | 2022.06.16 | Guidelines on the Issuance of Treatment Guidelines for Severe Acute Hepatitis in Children with Unexplained Causes (Trial) | Hepatitis B | Prevention | National Health Commission of PRC, National Disease Control and Prevention Administration. | Implementation document |
| 61 | 2022.07.29 | Interpretation on Catalogue of Industries for Encouraging Foreign Investment (2022 Edition) | General Liver Disease | Diagnosis and Treatment | National Disease Control and Prevention Administration. | Implementation document |
| 62 | 2022.08.20 | Guidelines for Liver Cancer Screening in the Chinese Population | General Liver Cancer | Early Detection | National cancer center | Implementation document |
| 63 | 2022.12.30 | Circular on the issuance of the Action Plan for the Elimination of Mother-to-Child Transmission of HIV, Syphilis and Hepatitis B (2022-2025) | Hepatitis B | Prevention | General Office of National Health Commission of PRC. | Official document |
| 64 | 2022.12.30 | the Clinical Application Guidelines for New Antineoplastic Drugs (2022 Edition) | Liver Cancer | Diagnosis and Treatment | National Health Commission of PRC | Implementation document |
| 65 | 2022.12.31 | Quality control index for standardized diagnosis and treatment of primary liver cancer in China (2022 edition) | General Liver Cancer | Diagnosis and Treatment | National Development and Reform Commission of PRC, Ministry of Commerce of PRC. | Implementation document |
| 66 | 2023.01.18 | China's National Basic Medical Insurance, Work Injury Insurance and Maternity Insurance (2022) | General Liver Disease | Prevention, Diagnosis and Treatment | National Health Commission of PRC, National Development and Reform Commission of PRC, Ministry of Education of PRC, Ministry of Science and Technology of PRC, Ministry of Civil Affairs of PRC, Ministry of Finance of PRC, Ministry of Ecology and Environment of PRC, Ministry of Agriculture and Rural Affairs of PRC, National Financial Regulatory Administration, National Healthcare Security Administration, National Administration of Traditional Chinese Medicine, National Disease Control and Prevention Administration, National Medical Products Administration. | Implementation document |
| 67 | 2023.03.07 | Specialized Quality Control Indicators for Oncology (2023 Edition) | Liver Cancer | Diagnosis and Treatment | National Health Commission of PRC | Implementation document |
| 68 | 2023.07.11 | Circular of World Hepatitis Day 2023 Theme Publicity Activities | Hepatitis B | Prevention | National Healthcare Security Administration, Ministry of Human Resources and Social Security of PRC. | Implementation document |
| 69 | 2023.10.30 | Healthy China Initiative - Implementation Plan for Cancer Prevention and Control (2023-2030) | General Liver Disease | Prevention, Diagnosis and Treatment, Early Detection, Surveillance | National Health Commission of PRC, National Development and Reform Commission of PRC, Ministry of Education of PRC, Ministry of Science and Technology of PRC, Ministry of Civil Affairs of PRC, Ministry of Finance of PRC, Ministry of Ecology and Environment of PRC, Ministry of Agriculture and Rural Affairs of PRC, National Financial Regulatory Administration, National Healthcare Security Administration, National Administration of Traditional Chinese Medicine, National Disease Control and Prevention Administration, National Medical Products Administration. | Implementation document |
| 70 | 2023.12.13 | China's National Basic Medical Insurance, Work Injury Insurance and Maternity Insurance (2023) | General Liver Disease | Prevention, Diagnosis and Treatment | National Health Commission of PRC, National Development and Reform Commission of PRC, Ministry of Education of PRC, Ministry of Science and Technology of PRC, Ministry of Civil Affairs of PRC, Ministry of Finance of PRC, Ministry of Ecology and Environment of PRC, Ministry of Agriculture and Rural Affairs of PRC, National Financial Regulatory Administration, National Healthcare Security Administration, National Administration of Traditional Chinese Medicine, National Disease Control and Prevention Administration, National Medical Products Administration. | Implementation document |
| 71 | 2024.01.03 | the Clinical Application Guidelines for New Antineoplastic Drugs (2023 Edition) | Liver Cancer | Diagnosis and Treatment | National Health Commission of PRC | Implementation document |
| 72 | 2024.04.15 | Standard for diagnosis and treatment of primary liver cancer (2024 edition) | Primary Liver Cancer | Diagnosis and Treatment | National Health Commission of PRC. | Implementation document |
| 73 | 2024.11.28 | China's National Basic Medical Insurance, Work Injury Insurance and Maternity Insurance (2024) | General Liver Disease | Prevention, Diagnosis and Treatment | National Health Commission of PRC, National Development and Reform Commission of PRC, Ministry of Education of PRC, Ministry of Science and Technology of PRC, Ministry of Civil Affairs of PRC, Ministry of Finance of PRC, Ministry of Ecology and Environment of PRC, Ministry of Agriculture and Rural Affairs of PRC, National Financial Regulatory Administration, National Healthcare Security Administration, National Administration of Traditional Chinese Medicine, National Disease Control and Prevention Administration, National Medical Products Administration. | Implementation document |
| 74 | 2025.01.07 | the Clinical Application Guidelines for New Antineoplastic Drugs (2023 Edition) | Liver Cancer | Diagnosis and Treatment | National Health Commission of PRC | Implementation document |

Abbreviations: AIDS, Acquired Immune Deficiency Syndrome; DIP, Diagnosis-Intervention Packet; HBV, Hepatitis B Virus; HCV, Hepatitis C Virus; TCM, Traditional Chinese Medicine; PRC, the People's Republic of China.

References

1. Ministry of Health, People's Republic of China. Plan for the Prevention and Control of Viral Hepatitis.1984.04.18. Available at: https://www.pkulaw.com/chl/c1319a4924a29757bdfb.html?keyword=%E7%97%85%E6%AF%92%E6%80%A7%E8%82%9D%E7%82%8E%E9%98%B2%E6%B2%BB%E6%96%B9%E6%A1%88&way=listView.Accessed 2023-12-31.
2. Ministry of Health, People's Republic of China. Trial Measures for Immunization of Hepatitis B Blood-Derived Vaccine Nationwide. 1987.09.14. Available at: https://www.pkulaw.com/chl/5a7ac935de7761c1bdfb.html?keyword=%E5%85%A8%E5%9B%BD%E4%B9%99%E5%9E%8B%E8%82%9D%E7%82%8E%E8%A1%80%E6%BA%90%E7%96%AB%E8%8B%97%E5%85%8D%E7%96%AB%E6%8E%A5%E7%A7%8D%E8%AF%95%E
3. Ministry of Health, People's Republic of China. Implementation Plan for Hepatitis B Vaccination Nationwide. 1991.10.25. Available at: https://www.pkulaw.com/chl/a8c26a7911c4371ebdfb.html?keyword=%E5%85%A8%E5%9B%BD%E4%B9%99%E8%82%9D%E7%96%AB%E8%8B%97%E6%8E%A5%E7%A7%8D%E5%AE%9E%E6%96%BD%E6%96%B9%E6%A1%88&way=listView. Accessed 2023-12-31.
4. Ministry of Health, People's Republic of China. Circular on Further Strengthening the Current Prevention and Control of Hepatitis .1993.08.16. Available at: https://www.pkulaw.com/chl/365405fe053a0766bdfb.html?keyword=%E5%8D%AB%E7%94%9F%E9%83%A8%E5%85%B3%E4%BA%8E%E8%BF%9B%E4%B8%80%E6%AD%A5%E5%8A%A0%E5%BC%BA%E5%BD%93%E5%89%8D%E8%82%9D%E7%82%8E%E9%98%B2
5. Ministry of Health, People's Republic of China. Circular on Printing and Distributing the Tenth Five-Year Plan Outline for Health Work .2001.07.23. Available at: http://www.nhc.gov.cn/guihuaxxs/s3585u/200107/231189af60ac4b99b4fc4aaac4aa1437.shtml.Accessed 2023-12-22.
6. Ministry of Health, People's Republic of China, Ministry of Finance, People's Republic of China. Circular on Including Hepatitis B Vaccine in Children's Immunization Program .2001.12.05. Available at: https://www.pkulaw.com/chl/f79edb73752bb767bdfb.html?keyword=%E5%85%B3%E4%BA%8E%E5%B0%86%E4%B9%99%E8%82%9D%E7%96%AB%E8%8B%97%E7%BA%B3%E5%85%A5%E5%84%BF%E7%AB%A5%E8%AE%A1%E5%
7. Ministry of Health, People's Republic of China. Outline of the Tenth Five-Year Plan and the 2010 Long-Term Plan for the Development of Health Science and Technology in China .2001.12.13. Available at: http://www.nhc.gov.cn/qjjys/s3577/200804/f53d584326624ece83e4f9f43cd9f5ae.shtml.Accessed 2023-12-25.
8. Ministry of Health, People's Republic of China. Outline of the Plan for Cancer Prevention and Control in China (2004-2010) .2004.02.25Bulletin of Chinese Cancer.2004;(02):3-6.
9. Ministry of Health, People's Republic of China. Guiding Opinions on the Training Outline for Rural Health Workers .2005.03.21. Available at: http://www.nhc.gov.cn/wjw/gfxwj/201304/e48517b382b04ba0ab6b67f1a9ead69a.shtml.Accessed 2023-12-25.
10. The State Council. Outline of the National Medium- and Long-Term Science and Technology Development Plan (2006-2020) .2005.12.29. Available at: https://www.gov.cn/gongbao/content/2006/content_240244.htm.Accessed 2023-12-25.
11. Ministry of Health, People's Republic of China. National Plan for the Prevention and Control of Hepatitis B (2006-2010) .2006.01.28. Available at: https://www.gov.cn/gzdt/2006-02/13/content_187608_2.htm.Accessed 2023-12-31.
12. Ministry of Health, People's Republic of China. Key Points of Knowledge for Publicity and Education on Prevention and Control of Hepatitis B .2006.09.02. Available at: http://www.nhc.gov.cn/wjw/jbyfykz/201304/4a74483709f34ec6ae87a0351e5af3e2.shtml.Accessed 2023-12-25.
13. Ministry of Health, People's Republic of China. Management Measures for Early Diagnosis and Treatment of Cancer Projects (Trial) .2007.01.23. Available at: https://www.pkulaw.com/chl/6ca1c2854a2cb25ebdfb.html?keyword=%E7%99%8C%E7%97%87%E6%97%A9%E8%AF%8A%E6%97%A9%E6%B2%BB%E9%A1%B9%E7%9B%AE%E7%AE%A1%E7%90%86%E5%8A%9E%E6%B3%95%EF%BC%88%E8%AF%95%E8%A1%8C%
14. Ministry of Health, People's Republic of China. Circular on Printing and Distributing the Implementation Plan for Expanding the National Immunization Program .2008.05.02. Available at: http://www.nhc.gov.cn/bgt/pw10803/200805/06e538cad856458bba92730b81e0f29c.shtml.Accessed 2023-12-31.
15. Ministry of Health, People's Republic of China. Circular on Printing and Distributing the Basic Responsibilities of Disease Prevention and Control Institutions at All Levels and the Performance Evaluation Standards for Disease Prevention and Control Work.2008.12.01. Available at: http://www.nhc.gov.cn/jkj/s7914g/200812/a9949418dac742239caac4332cbc3275ml.Accessed 2023-12-25.
16. Ministry of Health, People's Republic of China. Circular on Establishing the National Traditional Chinese Medicine Clinical Research Base TCM Prevention and Treatment of Liver Disease Clinical Research Alliance.2008.12.15. Available at: http://www.natcm.gov.cn/guicaisi/gongzuodongtai/2018-03-24/2186.html.Accessed 2023-12-25.
17. General Office of Ministry of Health, People's Republic of China. Circular on Carrying out the Standardized Diagnosis and Treatment Training Program for Chronic Hepatitis B in County-Level Hospitals. 2010.07.16.Available at: https://www.pkulaw.com/chl/c2526b13c2bbd290bdfb.html?keyword=%E5%85%B3%E4%BA%8E%E5%BC%80%E5%B1%95%E5%8E%BF%E7%BA%A7%E5%8C%BB%E9%
18. General Office of Ministry of Health, People's Republic of China. Guidelines for the Standardized Diagnosis and Treatment of Common Tumors in Municipal and County-Level Hospitals (Trial) .2010.12.14. Available at: http://www.nhc.gov.cn/yzygj/s3590/201012/325fdbaf4f46402680518dab22665760.shtml.Accessed 2023-12-25.
19. General Office of Ministry of Health, People's Republic of China. Circular on Printing and Distributing the Implementation Plan for the Prevention of Mother-to-Child Transmission of AIDS, Syphilis and Hepatitis B .2011.02.24. Available at: https://www.gov.cn/zwgk/2011-02/24/content_1809480.htm.Accessed 2023-12-25.
20. National People's Congress. Outline of the Twelfth Five-Year Plan for National Economic and Social Development of the People's Republic of China .2011.03.16. Available at: https://www.gov.cn/zhuanti/2011-03/16/content_2623428.htm.Accessed 2023-12-25.
21. Ministry of Health, People's Republic of China, Ministry of Finance, People's Republic of China, Ministry of Commerce, People's Republic of China, National Administration of Traditional Chinese Medicine. National Basic Public Health Service Specification (2011 Edition). 2011.05.24. Available at: https://www.gov.cn/zwgk/2011-05/24/content_1870181.htm.Accessed 2023-12-25.
22. Ministry of Health, People's Republic of China. Circular on Carrying out the 2011 World Hepatitis Day Publicity Activities .2011.07.12. Available at: https://www.gov.cn/gzdt/2011-07/12/content_1904494.htm. Accessed 2023-12-31.
23. National Administration of Traditional Chinese Medicine. Guidelines for the Construction and Management of Liver Disease Departments in Traditional Chinese Medicine Hospitals and Other Five Departments (Trial) .2012.02.22. Available at: http://www.natcm.gov.cn/yizhengsi/gongzuodongtai/2018-03-24/2924.html. Accessed 2023-12-25.
24. Ministry of Health, People's Republic of China. Circular on Organizing and Carrying out the 2012 World Hepatitis Day Publicity Activities .2012.07.06.Available at: https://www.gov.cn/gzdt/2012-07/06/content_2178093.htm.Accessed 2023-12-25.
25. The State Council, People's Republic of China. Circular on Printing and Distributing the Twelfth Five-Year Plan for the Development of Health Undertakings .2012.10.08. Available at: https://www.gov.cn/zhengce/zhengceku/2012-10/19/content_6074.htm.Accessed 2023-12-31.
26. Ministry of Health, People's Republic of China. Management Measures for Urban Cancer Early Diagnosis and Early Treatment Projects .2012.10.26.Available at: http://www.nhc.gov.cn/cms-search/xxgk/getManuscriptXxgk.htm?id=56178.Accessed 2023-12-25.
27. General Office of Ministry of Health, People's Republic of China. Evaluation Standards for the Construction of National Clinical Key Specialties (Trial) .2012.11.08Available at: https://www.pkulaw.com/chl/4a67ed0490996988bdfb.html?keyword=2011%E8%82%BF%E7%98%A4%E7%A7%91%E5%9B%BD%E5%AE%B6%E4%B8%B4%E5%BA%8A%E9%87%8D%E7%82%B9%E4%B8%93%E7%A7%91%E5%BB%BA%E
28. Ministry of Health, People's Republic of China. Quality Control Indicators for the Diagnosis and Treatment of Primary Liver Cancer (Trial) .2012.12.14Available at: https://www.pkulaw.com/chl/1ce59ab7fdaf7b5fbdfb.html?keyword=%E5%8E%9F%E5%8F%91%E6%80%A7%E8%82%9D%E7%99%8C%E8%AF%8A%E7%96%97%E8%B4%A8%E9%87%8F%E6%8E%A7%E5%88%B6%E6%8C%87%E6%A0%87%EF%BC%88%E
29. National Administration of Traditional Chinese Medicine, Ministry of health, Ministry of Education, People's Republic of China. Standardized Training Standards for General Practitioners of Traditional Chinese Medicine (Trial) .2013.02.17. Available at: https://www.pkulaw.com/chl/19434e080833aaf4bdfb.html?keyword=%E4%B8%AD%E5%8C%BB%E7%B1%BB%E5%88%AB%E5
30. National Health and Family Planning Commission, People's Republic of China, National Administration of Traditional Chinese Medicine. Circular on Printing and Distributing the Work Plan for Comprehensively Improving the Comprehensive Capability of County-Level Hospitals .2014.08.07.Available at: https://www.pkulaw.com/chl/ee1b64acfbe22f4abdfb.html?keyw
31. National Administration of Traditional Chinese Medicine, National Health and Family Planning Commission, People's Republic of China, Ministry of Education, People's Republic of China. Implementation Measures for the Standardized Training of Traditional Chinese Medicine Residents (Trial) .2014.12.11Available at: http://www.natcm.gov.cn/renjiaosi/zhengcewenjian/2018-03-24/1
32. Ministry of Health, People's Republic of China. Circular on Fully Implementing the Prevention of Mother-to-Child Transmission of AIDS, Syphilis and Hepatitis B .2015.06.16. Available at: http://www.nhc.gov.cn/fys/gzbs/201506/4f2123fa955a44afa75a75da2ad35d6e.shtml. Accessed 2023-12-25.
33. National Health and Family Planning Commission, People's Republic of China, National Development and Reform Commission, People's Republic of China, Ministry of Education, People's Republic of China. Three-Year Action Plan for Cancer Prevention and Control in China (2015-2017) .2015.09.09.Available at: https://www.pkulaw.com/chl/e478f4447cab158cbdfb.html?keyword=%E4%B8%AD%E5%9B%BD%E7%99%8C%E7%
34. General Office of National Health and Family Planning Commission, People's Republic of China. Circular on Printing and Distributing the Training Outline for Newly Recruited Nurses (Trial) .2016.02.16. Available at: http://www.nhc.gov.cn/yzygj/s3593/201602/91b5a8fa3c9a45859b036558a5073875.shtml.Accessed 2023-12-25.
35. National People's Congress. Outline of the Thirteenth Five-Year Plan for National Economic and Social Development of the People's Republic of China .2016.03.17. Available at: https://www.gov.cn/xinwen/2016-03/17/content_5054992.htm. Accessed 2023-12-31.
36. The Communist Party of China (CPC) Central Committee, the State Council. Outline of the "Healthy China 2030" Plan .2016.10.25. Available at: http://big5.www.gov.cn/gate/big5/www.gov.cn/zhengce/2016-10/25/content_5124174.htm. Accessed 2023-12-25.
37. The State Council. Circular on Printing and Distributing the "Thirteenth Five-Year" Deepening Medical and Health System Reform Plan .2016.12.27. Available at: https://www.gov.cn/zhengce/zhengceku/2017-01/09/content_5158053.htm. Accessed 2023-12-25.
38. The State Council. Circular on Printing and Distributing the "Thirteenth Five-Year" Health and Health Plan .2016.12.27. Available at: https://www.gov.cn/zhengce/zhengceku/2017-01/10/content_5158488.htm. Accessed 2023-12-25.
39. The State Council. Special Plan for Health and Health Science and Technology Innovation during the "Thirteenth Five-Year Plan" Period .2017.01.11. Available at: http://www.nhc.gov.cn/mohwsbwstjxxzx/s2908/201701/4dee1418b89548bca69e0dbf1652e1fd.shtml.Accessed 2023-12-25.
40. National Health and Family Planning Commission, People's Republic of China, National Development and Reform Commission, People's Republic of China, Ministry of Education, People's Republic of China, et al. China Viral Hepatitis Prevention and Control Plan (2017-2020) .2017.10.17. Available at: https://www.gov.cn/gongbao/content/2018/content_5271799.htm.Accessed 2023-12-31.
41. National Health Commission, People's Republic of China. Circular on Publishing 7 Health Industry Standards Including "Diagnosis of Hepatitis C" .2018.03.28. Available at: http://www.nhc.gov.cn/fzs/s7852d/201803/f78712f30cd04c08b53acfdcc1998562.shtml.Accessed 2023-12-25.
42. National Healthcare Security Administration. Circular on Carrying out Provincial-Level Centralized Procurement of Anti-Cancer Drugs. 2018.07.17. Available at: https://www.nhsa.gov.cn/art/2018/7/17/art_53_1158.html.Accessed 2023-12-31.
43. General Office of the State Council. Circular on Printing and Distributing the Key Tasks of Deepening Medical and Health System Reform in the Second Half of 2018 .2018.08.20. Available at: https://www.gov.cn/gongbao/content/2018/content_5319818.htm.Accessed 2023-12-25.
44. National Health Commission, People's Republic of China. the Clinical Application Guidelines for New Antineoplastic Drugs (2018 Edition). 2018.09.21. Available at: http://www.nhc.gov.cn/yzygj/s7659/201809/0ea15475f58a4f36b675cfa4716fa1e4.shtml. Accessed 2023-12-25.
45. National Healthcare Security Administration. Circular from the National Medical Security Administration on Including 17 Anti-Cancer Drugs in the Category B List of National Basic Medical Insurance, Work Injury Insurance and Maternity Insurance Drug Catalogue .2018.09.30. Available at: https://www.nhsa.gov.cn/art/2018/10/10/art_104
46. National Health Commission, People's Republic of China, National Administration of Traditional Chinese Medicine.2018 Edition of the National Essential Drug List .2018.10.15. Available at: http://www.nhc.gov.cn/yaozs/s7656/201810/c18533e22a3940d08d996b588d941631.shtml.Accessed 2023-12-25.
47. The State Council. Opinions of the State Council on Implementing the "Healthy China Initiative" .2019.07.15. Available at: https://www.gov.cn/zhengce/content/2019-07/15/content_5409492.htm.Accessed 2023-12-25.
48. National Health Commission, People's Republic of China, National Development and Reform Commission, People's Republic of China, Ministry of Education, People's Republic of China, et al. Healthy China Initiative - Implementation Plan for Cancer Prevention and Control (2019-2022) .2019.09.20. Available at: https://www.gov.cn/zhengce/zhengceku/2019-11/13/content_5451694.htm. Accessed 2023-12-25.
49. National Health Commission, People's Republic of China. the Clinical Application Guidelines for New Antineoplastic Drugs (2019 Edition). 2019.12.20. Available at: http://www.nhc.gov.cn/yzygj/s7659/201912/3922e93c3ef84c54879f36777db73568.shtml. Accessed 2023-12-31.
50. National Healthcare Security Administration. Circular from the Office of the National Medical Security Administration on Printing and Distributing the Detailed Grouping Scheme for Disease Diagnosis Related Groups (CHS-DRG) (Version 1.0) .2020.06.18. Available at: https://www.nhsa.gov.cn/art/2020/6/18/art_37_3240.html?ivk_sa=102432
51. National Medical Products Administration, National Health Commission, People's Republic of China. 2020 Edition of the "Pharmacopoeia of the People's Republic of China" .2020.07.12. Available at: https://www.nmpa.gov.cn/xxgk/ggtg/ypggtg/ypqtggtg/20200702151301219.html.Accessed 2023-12-25.
52. Office of National Healthcare Security Administration. Circular on the Issuance of the National Health Insurance Fractional Payment by Disease (DIP) Technical Specifications and the DIP Disease Catalog Library (Version 1.0).2020.11.20. Available at: https://www.nhsa.gov.cn/art/2020/11/20/art_37_3987.html.Accessed 2023-12-25.
53. National Health Commission, People's Republic of China. the Clinical Application Guidelines for New Antineoplastic Drugs (2020 Edition). 2020.12.30. Available at: http://www.nhc.gov.cn/yzygj/s7659/202012/6c00e8559ee54cd29585c7f39e8a23c4.shtml. Accessed 2023-12-31.
54. National Healthcare Security Administration, Ministry of Finance, People's Republic of China. Circular on Accelerating Cross-Provincial Direct Settlement of Outpatient Expenses .2021.04.12. Available at: https://www.gov.cn/zhengce/zhengceku/2021-05/06/content_5604906.htm. Accessed 2023-12-31.
55. Office of National Healthcare Security Administration, General Office of Ministry of Finance, People's Republic of China. Circular on the Pilot Work of Cross-Provincial Direct Settlement of Treatment Costs Related to Outpatient Chronic and Special Diseases .2021.09.14. Available at: https://www.nhsa.gov.cn/art/2021/9/14/art_53_5957.html.Accessed 2023-
56. General Office of National Health Commission, People's Republic of China, General Office of Ministry of Science and Technology, People's Republic of China, General Office of Ministry of Industry and Information Technology, People's Republic of China, et al. Circular on Printing and Distributing the Action Plan for Eliminating Public Health Hazards of Hepatitis C (2021-2030) .2021.09.15. Avail
57. General Office of National Health Commission, People's Republic of China. Circular on the Issuance of Criteria for the Establishment of National Cancer Medical Centers .2021.11.03. Available at: http://www.nhc.gov.cn/yzygj/s3594q/202111/b84b44c1ad9f474a89de34b3748b872b.shtml.Accessed 2023-12-25.
58. General Office of National Health Commission, People's Republic of China. Standard for diagnosis and treatment of primary liver cancer (2022 edition) .2022.01.21.Available at: http://www.nhc.gov.cn/yzygj/s7659/202201/a01ceb75c62b486fa459e36ba0fdfdbc.shtml.Accessed 2023-12-25.
59. General Office of the State Council. The 14th Five-Year National Health Plan. 2022.04.27. Available at: https://www.gov.cn/zhengce/content/2022-05/20/content_5691424.htm.Accessed 2023-12-25.
60. General Office of National Health Commission, People's Republic of China. Guidelines on the Issuance of Treatment Guidelines for Severe Acute Hepatitis in Children with Unexplained Causes (Trial).2022.06.16. Available at: http://www.nhc.gov.cn/yzygj/s7653p/202206/2765dbe6609e4580b9859a73fdb8fa14.shtml.Accessed 2023-12-31.
61. National Development and Reform Commission, People's Republic of China, Ministry of Commerce, People's Republic of China. Interpretation on Catalogue of Industries for Encouraging Foreign Investment (2022 Edition) .2022.07.29. Available at: https://www.gov.cn/zhengce/2022-11/29/content_5730383.htm.Accessed 2023-12-31.
62. He J, Chen WQ, Shen HB, et al. China guideline for liver cancer screening (2022, Beijing). Journal of Clinical Hepatology. 2022;38(08):1739-1758+1954-1967.
63. National Health Commission, People's Republic of China. Circular on the issuance of the Action Plan for the Elimination of Mother-to-Child Transmission of HIV, Syphilis and Hepatitis B (2022-2025).2022.12.30. Available at: http://www.nhc.gov.cn/fys/s3581/202212/afe6bc9626be45a0b25bee93f01fef10.shtml.Accessed 2023-12-25.
64. National Cancer Center, Liver Cancer Expert Committee of National Cancer Quality Control Center. Quality control index for standardized diagnosis and treatment of primary liver cancer in China (2022 edition). Electron J of Liver Tumor. 2022;9(04):1-11.
65. National Health Commission, People's Republic of China. the Clinical Application Guidelines for New Antineoplastic Drugs (2022 Edition). 2022.12.30. Available at: http://www.nhc.gov.cn/yzygj/s7659/202212/8df034c9afb44a9d95cd986d4e12fbd8.shtml. Accessed 2023-12-31.
66. National Healthcare Security Administration, Ministry of Human Resources and Social Security, People's Republic of China. China's National Basic Medical Insurance, Work Injury Insurance and Maternity Insurance (2022) .2023.01.18. Available at: https://www.nhsa.gov.cn/art/2023/1/18/art_14_10082.html.Accessed 2023-12-31.
67. National Health Commission, People's Republic of China. Specialized Quality Control Indicators for Oncology (2023 Edition). 2023.03.07. Available at: http://www.nhc.gov.cn/yzygj/s7657/202303/d61a0abf132f4aaf9ebbb6d094764ad2.shtml. Accessed 2023-12-25.
68. National Disease Control and Prevention Administration. Circular of World Hepatitis Day 2023 Theme Publicity Activities .2023.07.11. Available at: https://www.ndcpa.gov.cn/jbkzzx/c100014/common/content/content_1678934458566569984.html. Accessed 2023-12-25.
69. National Health Commission, People's Republic of China, National Development and Reform Commission, People's Republic of China, Ministry of Education, People's Republic of China, at al. Healthy China Initiative - Implementation Plan for Cancer Prevention and Control (2023-2030) .2023.10.30. Available at: https://www.gov.cn/zhengce/zhengceku/202311/content_6915380.htm.Accessed 2023-12-25.
70. National Healthcare Security Administration, Ministry of Human Resources and Social Security, People's Republic of China. China's National Basic Medical Insurance, Work Injury Insurance and Maternity Insurance (2023) .2023.12.13. Available at: https://www.nhsa.gov.cn/art/2023/12/13/art_104_11673.html.Accessed 2023-12-31.
71. National Health Commission, People's Republic of China. the Clinical Application Guidelines for New Antineoplastic Drugs (2023 Edition). 2024.01.03. Available at: http://www.nhc.gov.cn/yzygj/s7659/202401/48be6030a029446c93b852bfd4a5fdf6.shtml. Accessed 2024-12-31.
72. General Office of National Health Commission, People's Republic of China. Standard for diagnosis and treatment of primary liver cancer (2024 edition). 2024.04.15. Available at: http://www.nhc.gov.cn/yzygj/s7659/202201/a01ceb75c62b486fa459e36ba0fdfdbc.shtml. Accessed 2024-12-31.
73. National Healthcare Security Administration, Ministry of Human Resources and Social Security, People's Republic of China. China's National Basic Medical Insurance, Work Injury Insurance and Maternity Insurance (2024). 2024.11.28. Available at: http://www.nhc.gov.cn/yzygj/s7659/202404/653069140ddb4df28cdeba1ff1b86c66.shtml. Accessed 2023-12-31.
74. National Health Commission, People's Republic of China. the Clinical Application Guidelines for New Antineoplastic Drugs (2023 Edition). 2025.01.07. Available at: http://www.nhc.gov.cn/yzygj/s7659/202501/c1b6a86e2e6040aca75fdf89bc382184.shtml. Accessed 2024-12-31.
